# Stability of Propofol (2,6-Diisopropylphenol) in Thermal Desorption Tubes during Air Transport

**DOI:** 10.1155/2019/3987417

**Published:** 2019-05-02

**Authors:** Felix Maurer, Martin Geiger, Thomas Volk, Daniel I. Sessler, Sascha Kreuer, Tobias Hüppe

**Affiliations:** ^1^CBR-Center of Breath Research, Department of Anaesthesiology, Intensive Care and Pain Therapy, Saarland University Medical Center and Saarland University Faculty of Medicine, 66424 Homburg/Saar, Germany; ^2^Department of Outcomes Research, Anesthesiology Institute, Cleveland Clinic, Cleveland, OH, USA

## Abstract

The anesthetic propofol and other exhaled organic compounds can be sampled in Tenax sorbent tubes and analyzed by gas chromatography coupled with mass spectrometry. The aim of this study was to evaluate the stability of propofol in Tenax sorbent tubes during overseas shipping. This is relevant for international pharmacokinetic studies on propofol in exhaled air. Tenax sorbent tube propofol samples with concentrations between 10 and 100 ng were prepared by liquid injection and with a calibration gas generator. For each preparation method, one reference set was analyzed immediately after preparation, a second set was stored at room temperature, and a third one was stored refrigerated. The fourth set was sent from Germany by airmail to USA and back. The shipped set of tubes was analyzed when it returned after 55 days elapsed. Then, the room temperature samples and the refrigerated stored samples were also analyzed. To evaluate the stability of propofol in the stored and shipped tubes, we calculated the recovery rates of each sample set. The mean recovery in the stored samples was 101.2% for the liquid preparation and 134.6% for the gaseous preparation at 4°C. At 22°C, the recovery was 96.1% for liquid preparation and 92.1% for gaseous preparation, whereas the shipped samples had a recovery of 85.3% and 111.3%. Thus, the deviation of the shipped samples is within a range of 15%, which is analytically acceptable. However, the individual values show significantly larger deviations of up to -32.1% (liquid) and 30.9% (gaseous). We conclude that storage of propofol on Tenax tubes at room temperature for 55 days is possible to obtain acceptable results. However, it appears that due to severe temperature and pressure variations air shipment of propofol samples in Tenax tubes without cooling shows severe deviations from the initial concentration. Although it was not tested in this study, we assume that refrigerated transport might be necessary to obtain comparable results as in the stored samples.

## 1. Introduction

Propofol is one of the most commonly used intravenous anesthetics. Due to the high volume of distribution and the high clearance, relatively high plasma concentrations are required for adequate anesthesia. However, there is no fast method for determining the plasma concentration of the agent during anaesthesia. Since propofol is volatile and the blood and alveolar space are in equilibrium after a defined time, the drug is exhaled during anesthesia [1]. Several studies have shown a good correlation between the exhaled and plasma propofol concentration [2, 3].

Online measurement of propofol concentrations in exhaled air is an innovative approach for personalized dosage. The opportunity for this promising technique has now emerged due to a new mobile ion mobility spectrometry device (EDMON, Exhaled Drug Monitor; B. Braun Melsungen, Melsungen, Germany) on the market. Worldwide studies are expected to establish pharmacological models for the prediction of propofol concentrations in blood from breath concentration since a direct inference from the breath to the blood level is not possible as exhaled concentrations adapt to changes in the plasma concentration with a time delay when concentrations are not in steady state [4].

Nevertheless, the gold standard for such analyses is the gas chromatography with coupled mass spectrometer (GCMS). It serves as a reference method for other methods such as the ion mobility spectrometry. Therefore, in addition to IMS measurements, GCMS measurements are also necessary to allow cross-validation of the results. A GCMS with thermal desorption system (TDS) allows direct sampling of exhaled air on Tenax sorbent tubes and quantification without further sample preparation [5]. However, the method requires expensive equipment and considerable expertise. Therefore, TDS-GCMS systems are only available in specialized centers, so shipping of patient samples is unavoidable.

In a recent study, we demonstrated that propofol is storable on Tenax sorbent tubes for at least two weeks at ambient temperature [6]. Overseas shipping, however, often takes longer and is characterized by significant variations in atmospheric pressure and temperature, as well as strong vibrations. It is unknown whether propofol concentrations remain stable during air shipments. We, thus, tested the hypothesis that propofol concentrations in Tenax tubes remain stable when sent by airfreight from Germany to the United States and back.

## 2. Materials and Methods

### 2.1. Sample Preparation with a Stock Solution

We dissolved propofol (97%, Sigma Aldrich, Munich, Germany) in HPLC-grade water (VWR, Darmstadt, Germany) to a 50 *µ*g/mL propofol stock solution. The stock solution was gravimetrically diluted in 50 mL flasks to 5, 10, 20, and 50 *µ*g/mL samples. 2 *µ*L from each standard or HPLC-grade water for blanks, respectively, was directly pipetted onto Tenax sorbent tubes as quadruplicate per sample. The mass of each injection was determined on a Cubis analytical scale (Sartorius, Göttingen, Germany) to determine the exact amount of propofol injected in ng. The resulting samples contained final propofol masses of 10, 20, 50, and 100 ng. For an even distribution of propofol, each tube was flushed after injection for 30 seconds with 1 bar of 99.999% pure synthetic air (Air Liquide, Düsseldorf, Germany) (20.5% O_2_ purity [4.5], 79.5 % N_2_ purity [5.0]). To test whether propofol is lost during loading, a second Tenax sorption tube was used to investigate the synthetic air exiting the end of the loaded tube. We calibrated on the day of shipment and immediately after their return, with eight calibration standards (0, 10, 20, 30, 40, 50, 60, and 100 ng) prepared on the day of calibration, which were pipetted as stated above.

### 2.2. Sample Preparation with Calibration Gas Generator

A 90 *µ*g/mL propofol stock solution was prepared as described [6] with addition of 1% v/v HPLC-grade ethanol (Sigma-Aldrich GmbH, Steinheim, Germany). The gas generator was a HovaCAL 4836-VOC (IAS GmbH, Oberursel, Germany) [7]. The propofol stock solution was injected via two syringes (1702.5TLLX, Hamilton Co., Reno, USA), each with a volume of 50 *µ*L and vaporized at 100°C. The Tenax sorbent tubes were loaded by aspirating 0.3 L propofol gas with a flow of 0.4 L*∙*min^−1^ through a Bivoc2 gas sampling pump (Holbach GmbH, Wadern, Germany).

Tenax sorbent tubes with a total amount of 20, 40, 60, 80, and 100 ng of vaporized propofol were prepared as quadruplicate per sample. After every change of the propofol output concentration, the reference gas generator was operated for 5 minutes before sampling to ensure the equilibration at the designated concentration.

We calibrated on the day of shipment and immediately after their return, with five calibration standards (20, 40, 60, 80, and 100 ng) prepared on the day of calibration, which were prepared as mentioned above. The sampling method in detail and HovaCAL parameters are mentioned elsewhere [6]. The agreement between both sampling preparation methods is mentioned elsewhere [6].

### 2.3. Storage and Shipment of Samples

Each sample was prepared as quadruplicate on the day of shipment and randomly assigned to one of four groups. The reference samples were analyzed immediately after their preparation. Two sets of samples were stored at 21°C and 4°C, respectively, and analyzed after 55 days. The mailed samples were sent to Cleveland, Ohio, USA, and analyzed upon return after 55 days.

Every TDS-Tube was stored in its respective tube container and an overpack. A data logger (Log32THP, Dostmann, Wertheim, Germany) was added to the stored and shipped samples to record temperature and pressure variations in the interval of five minutes. The amounts for the reference samples were calculated with the calibration at the beginning of the experiment. Amounts for stored and mailed samples were determined with calibration at the end of the experiment.

### 2.4. GC-MS

We quantified propofol using a 7890B gas chromatograph (Agilent, Santa Clara, United States) with a 5977B quadrupole mass spectrometer (Agilent, Santa Clara, United States) and an XTr EI 350 ion extraction lens. For sample preparation and application into the GC-MS, we used Tenax sorbent tubes (Tenax GR, Gerstel, Mühlheim, Germany). A coupled TDSA2 auto sampler (Gerstel, Mühlheim, Germany) with a TDS3 thermal desorption system (Gerstel, Mühlheim, Germany) and a KAS6 cold injection system (Gerstel, Mühlheim, Germany) was used for injection. The carrier gas was helium (99.9999%, Air Liquide Deutschland GmbH, Düsseldorf, Germany). For separation we used a capillary column of the type HP-5MS UI (30 m x 0.25 mm, film thickness 0.25 *µ*m, Agilent Technologies, Santa Clara, United States) and a column flow of 1.2 mL/min. The column temperature program started at 50°C with a temperature ramp of 20 K/min and a final temperature of 260°C for 1 min. The thermal desorption program had a ramp rate of 20 K/min with a final temperature of 240°C for 4 min, unsplit sample, and a desorption flow 40 mL/min. The cold injection conditions were -50°C with a temperature ramp of 12 K/s and a final temperature of 250°C for 3 min, 20:1 injection split. The mass spectrometer conditions were full scan m/z between 50 and 300, transfer tube temperature 250°C, quadrupole temperature 150°C, ion source temperature 230°C, and solvent delay 4 min. Propofol has a retention time (RT) from 7,489 to 7,501 min in this setup [6].

### 2.5. Data Analysis

For data analysis, we used the programs Qualitative Analysis (ver. B.07.000) and Quantitative Analysis (ver. B.07.01 SP1) (Agilent Technologies). For peak identification, the NIST-Database was applied. As quantifier and qualifier ions, m/z 163 and m/z 178 were used. The percentage deviation, which defines the acceptable range for the quantifier to qualifier ratio, was set at 20%. The measured peak area of propofol was plotted vs. the known weighed masses of propofol, respectively, vs. known ppbv of propofol. Slope and intercept were determined from a least-squares linear regression. For the linear regression, the acceptable R^2^ minimum was set at 0.985.

The recovery was calculated according to the equation

(1) $$\begin{array}{c} \frac{\left(measured\;mass-weighed\;mass\right)*100}{measured\;mass} \end{array}$$

### 2.6. Statistical Analysis

SigmaPlot (version 12.5, Systat Software, Erkrath, Germany) was used for statistical analysis. A Bland and Altman analysis was performed to evaluate the agreement between different sample groups.

## 3. Results

### 3.1. Tenax Sorbent Tubes

**Figure 1:**
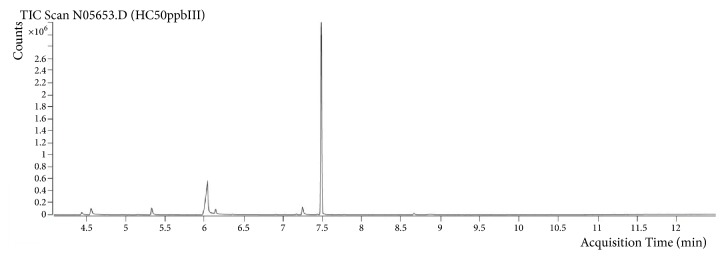
Example chromatogram for propofol (gaseous application; 50 ppb_v_).

Every tube yielded an analyzable chromatogram. No peak interferences were observed around the retention time of propofol (RT=7.5 min) (Figure 1). Besides the usual Tenax degradation products, such as phenol (RT = 4.5 min), acetophenone (RT = 5.3min), benzoic acid (RT = 6.1min), and phthalic anhydride (RT = 7.2 min) [8], propofol was always the only sharply demarcated signal.

The propofol quantifier peak (base peak 163 m/z) to qualifier peak (molecule peak 178 m/z) ratio has always met our analyzing criteria and gained a mean value of 29.4 ± 5% SD.

**Figure 2:**
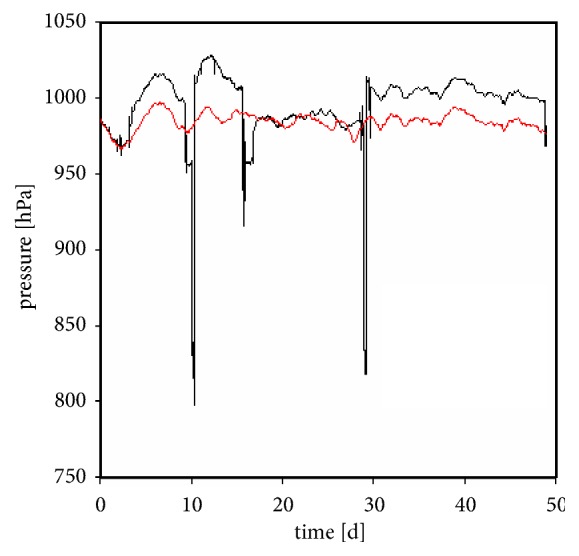
Pressure course for the stored samples (red line) and the mailed samples (black line).

Figure 2 shows the pressure course for both the stored and the mailed samples over the shipping time. Latter samples were transported on three flights with a minimum total pressure of ~800 hPa, which resembles ~80% of the normal atmospheric pressure. The stored samples were not subject to strong pressure fluctuations.

**Figure 3:**
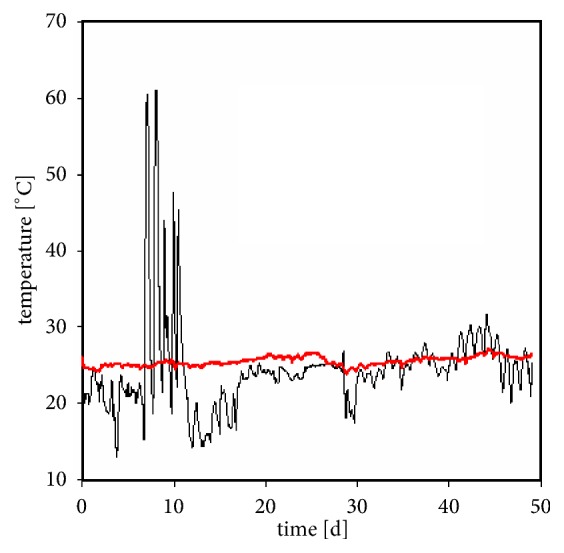
Temperature course for the stored samples (red line) and the mailed samples (black line).

Figure 3 shows the temperature course for stored and mailed samples. The mailed samples experienced some temperature spikes up to 60°C during transportation and periodical changes in temperature at around room temperature, which is likely due to the day-and-night cycles. The stored samples remained at ambient temperature throughout.

The measured amounts of propofol are shown in Table 1. The mailed samples had a mean recovery of 85.3% for the liquid preparation and 111.3% for the gaseous preparation. In contrast, the stored samples showed average recovery rates of 101.2% and 134.6% at 4°C and 96.1%, respectively, and 92.1% at 22°C. Figure 2 shows the differences between the stored and mailed samples, respectively, and the reference samples that were analyzed immediately.

**Figure 4:**
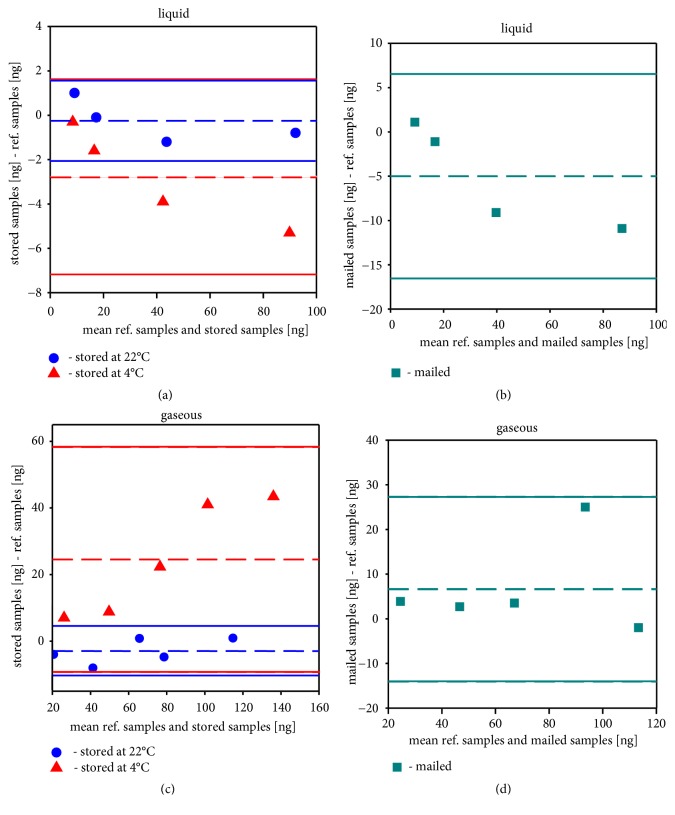
Comparison of stored (a, c) and mailed (b, d) samples with the reference Tenax tube samples for both application methods: liquid (a, b) and gaseous (c, d). Difference plots with stored samples (22°C blue dots; 4°C red triangles) vs. direct measured reference samples (a, c) and mailed samples vs. direct measured reference vs. (turquoise rectangles) (b, d). The dashed lines represent the mean of the differences and the solid lines represent the mean of the difference ± 1.96 SD.

The comparison of the mailed samples vs. reference samples (Figures 4(b) and 4(d)) shows a trend to negative differences for the liquid application at higher propofol concentrations, whereas the gaseous applicated samples show no tendency. At least, it is clear that the propofol is not lost during the loading process, since a second sorbent tube was used to confirm that the synthetic air escaping at the end of the tube does not contain any propofol.

The data for the chilled samples show a greater deviation than the samples stored at room temperature for both application methods (Figures 4(a) and 4(c)) and a significant overestimation of the propofol amount for the gaseous samples (Figure 4(c)).

## 4. Discussion

The total delivery time of Tenax sorption tubes was 55 days. Over this period, ambient air storage resulted in acceptable mean recovery rates for liquid applicated samples. However, airfreight shipping, on the other hand, was associated with a significant loss of propofol in liquid applied, but not in gaseous applied samples. Even storage of liquid applied samples at room temperature resulted in a much lower loss than air freight.

In principle, the differences in the recovery rates could be due to the storage time itself, on the one hand, and the pressure and temperature fluctuations during air transport, on the other hand. Naturally, a stronger diffusion and desorption rate is expected at higher propofol concentration on the tubes. The transported samples were repeatedly exposed to high temperatures of over 60°C. We can only speculate about the reasons. The samples may have been stored between flights in uncooled freight centers. Furthermore, low pressure during long-haul flights (~800 hPa and ~825 hPa) and vibrations may explain the loss of propofol during air transport. However, why this loss affects more strongly the liquid samples is unclear. It would be conceivable that the gaseous application leads to a more uniform distribution of the propofol on the surface of the adsorbent and thus to a better analyte retention than a liquid solution dropped locally on the Tenax. Whether that explains the differences is not clear, but the gaseous samples are more similar in their nature to real breath samples and therefore particularly important for evaluating durability during air transport. The liquid application method has the advantage that the mass of propofol can be weighed exactly on the tube. Therefore, it provides accurate control of the amount of propofol. For the gaseous application, this is clearly not possible, although this technique is less cumbersome and closer to the real breath sample.

Considering the recovery rate in the reference samples, it can be assumed that even without storage or shipping due to measurement inaccuracies and errors during loading and weighing of the samples a mean error of around ±7.5% can occur. With regard to our validated method inaccuracy [5] of ~ 6.5 RSD%, data within a range of 13% RSD are within the inaccuracy of the TDS-GCMS method since the comparison with the reference must be based on double the RSD due to inaccuracies of both the calibration standards and the samples. This may explain the recovery rates of the refrigerated samples which were almost all >100% despite a storage period of 55 days.

In a clinical setup, exhaled air concentrations in a range of 50 ppb_v_ (corresponding 110 ng) can be expected at maximum [6, 9]. As our own clinical measurements on patients during propofol anesthesia show, the measured concentrations in exhaled air are mostly between 0 and 22 ng, which is within the range we studied now. For the shipped tubes, however, there are samples with a difference of over 30% compared to the reference, which corresponds to 16 ng. Looking now at the relevant measuring range of 0-22 ng, these deviations are far outside the usual tolerances of 10-15%. This suggests that the air transport might be possible for exhaled air without affecting the propofol amount but is not possible with the approach we have chosen.

Nevertheless, the study has several limitations. Unfortunately, refrigerated air transport has not been tested for cost reasons, although chilled storage is published to have a more consistent analyte retention than uncooled storage [10]. Although the propofol loss in our experiments was also smaller for chilled samples, a more constant concentration could not be determined. Furthermore, the stability of propofol in Tenax tubes may also depend on the exhaled matrix itself. We cannot rule out that other volatile compounds affect the stability of propofol and the sampling technique might have an impact on the recovery as well.

## 5. Conclusions

Air shipment of propofol samples in Tenax tubes without cooling shows unacceptable deviations from the initial concentration and is, therefore, not recommended.

## Figures and Tables

**Table 1:** Given and measured propofol amounts of Tenax sorbent tubes. The column “target” shows the desired concentration per tube. The column “weighed” shows the calculated amounts of propofol per tube and “measured” shows the actual measured amount. The columns Δmeasured-weighed and Δmeasured-reference displays the percentual difference of the columns, with reference to the measured mass.

liquid												
	reference			samples stored at 4°C			samples stored at 22°C			mailed samples		
target	weighed	measured	Δmeasured- weighed	weighed	measured	Δmeasured-weighed	weighed	measured	Δmeasured-weighed	weighed	measured	Δmeasured-weighed
[ng]	[ng]	[ng]	%	[ng]	[ng]	%	[ng]	[ng]	%	[ng]	[ng]	%
10	9.8	8.6	-14.0	8.6	8.3	-3.6	9.6	9.6	0.0	9.5	9.7	2.1
20	18.5	17.3	-6.9	15.5	15.7	1.3	18.2	17.2	-5.8	18.1	16.2	-11.7
50	47.8	44.3	-7.9	38.3	40.4	5.2	45.5	43.1	-5.6	46.5	35.2	-32.1
100	96.4	92.5	-4.2	85.6	87.2	1.8	95.4	91.7	-4.0	95.4	81.6	-16.9

mean			-8.3			1.2			-3.9			-14.7

gaseous												
	reference			samples stored at 4°C			samples stored at 22°C			mailed samples		
target	measured			measured		Δmeasured- reference	measured		Δmeasured- reference	measured		Δmeasured- reference
[ng]	[ng]			[ng]		%	[ng]		%	[ng]		%

20	22.6			29.6		31.0	18.6		-17.7	26.5		17.3
40	45.3			54.1		19.4	37.2		-17.9	48.0		6.0
60	65.3			87.6		34.2	66.1		1.2	68.8		4.1
80	81.0			122.0		50.6	76.2		-5.9	106.0		30.9
100	114.3			157.7		38.0	115.2		0.8	112.3		-1.7

						34.6			-7.9			11.3

## Data Availability

All relevant data to support the findings of this study are included within the article.
